# 基于L-谷氨酸的手性硅胶球的制备及其应用

**DOI:** 10.3724/SP.J.1123.2020.07034

**Published:** 2021-06-08

**Authors:** Wanqi XIONG, Bo PENG, Aihong DUAN, Liming YUAN

**Affiliations:** 云南师范大学化学化工学院, 云南 昆明 650500; School of Chemistry and Chemical Engineering, Yunnan Normal University, Kunming 650500, China; 云南师范大学化学化工学院, 云南 昆明 650500; School of Chemistry and Chemical Engineering, Yunnan Normal University, Kunming 650500, China; 云南师范大学化学化工学院, 云南 昆明 650500; School of Chemistry and Chemical Engineering, Yunnan Normal University, Kunming 650500, China; 云南师范大学化学化工学院, 云南 昆明 650500; School of Chemistry and Chemical Engineering, Yunnan Normal University, Kunming 650500, China

**Keywords:** 高效液相色谱, 制备, 手性分离, 手性硅胶球固定相, high performance liquid chromatography (HPLC), preparation, enantioseparation, chiral silica gel spheres stationary phase

## Abstract

无机介孔硅球因其具有足够的机械强度、热稳定性,以及适应多种流动相的优点,成为高效液相色谱(HPLC)柱填料中使用最广泛和最重要的材料。但在此研究领域中,并未见球形的全无机手性硅胶用作HPLC手性固定相。该文以无机球形介孔硅胶作为研究对象,通过堆砌硅珠法,以硅溶胶为原料,L-谷氨酸(L-Glu)为手性源,在手性环境中制造出脲醛树脂与胶体二氧化硅混合的小球,在550 ℃高温下煅烧除去树脂部分,制备基于L-Glu的无机介孔硅胶球。通过元素分析、红外光谱、扫描电镜、透射电镜和氮气吸附等表征证明这是一种具有规则球形的手性硅胶球,其手性来源于硅胶球自身的骨架和孔结构。将L-Glu手性硅胶球作为固定相制备了HPLC色谱柱,以正己烷-异丙醇(9∶1, v/v)作为流动相,流速为0.1 mL/min,考察了该手性柱对一系列外消旋化合物的拆分性能。实验表明,该手性柱拆分了15种外消旋化合物,其中特罗格尔碱、吡喹酮、3-苄氧基-1,2-丙二醇、1,2-环氧己烷、3-羟基-2-丁酮、2-甲基四氢呋喃-3-酮、异丙基缩水甘油醚达到基线分离;还分离了10种苯系位置异构体,*o*,*m*,*p*-氨基苯酚、*o*,*p*-氯苯酚、*o*,*m*,*p*-碘苯胺、*o*,*m*,*p*-甲苯胺、*o*,*m*,*p*-二硝基苯、*o*,*m*,*p*-氯苯胺、*o*,*m*,*p*-硝基苯酚、*o*,*m*,*p*-溴苯胺达到基线分离。实验表明,L-Glu手性硅胶球在手性分离方面具有良好的可行性,与普通硅胶相比不需要进一步修饰就可以有较好的手性分离效果,是一种低成本、制备便捷的手性无机硅胶固定相。

## Abstract (en)

Inorganic mesoporous silica gel spheres, which possess sufficient mechanical strength, thermal stability, and mobile phase endurance, are the most important and widely used materials for column packing in high performance liquid chromatography (HPLC). However, amorphous silica gel is generally reported as an inorganic chiral silica gel, and spherical all-inorganic chiral silica gel has not been reportedly used as the chiral stationary phase for HPLC. In this paper, inorganic spherical mesoporous silica gel was used in the method of polymerization-induced colloid aggregation (PICA), with silica sol as the raw material and L-glutamic acid (L-Glu) as the chiral monomer, to obtain mixed spheres of urea-formaldehyde resin and colloidal silica in a chiral environment. After high-temperature calcination (at 550 ℃) to remove the resin, inorganic mesoporous silica gel spheres based on L-Glu were prepared. Elemental analysis revealed that the prepared L-Glu chiral silica gel spheres were calcined completely, indicating that there were no organic constituents. Scanning electron microscopy (SEM) images of the silica gel spheres showed that the surface of the silica gel spheres was not smooth, with a uniform particle size of 3.0-4.5 μm. Transmission electron microscopy (TEM) images showed that the pore size distribution of the synthetic silica gel spheres was uniform due to the accumulation of pores. Nitrogen adsorption tests revealed that the specific surface area of L-Glu chiral silica gel spheres was 117.844 m^2^/g, the pore volume was 0.411 cm^3^/g, and the average pore size was 12.312 nm. All the characterizations indicated that the inorganic chiral mesoporous silicon had a regular spherical shape. The silica gel spheres possess frameworks and pore structures, providing a chiral microenvironment that is suitable as a chiral stationary phase for separating racemic compounds by HPLC. Because of the chiral pore structure and frameworks, the enantiomers were retained to different degrees and separation was achieved. The porous structure also increased the contact surface between the racemates and the active sites of the inner wall and improved the separation efficiency. Hydrogen bonding between the chiral stationary phase and the racemates, dipole interactions, and van der Waals forces were also involved in enantiomer resolution. An HPLC column was prepared with L-Glu chiral silica gel as the stationary phase and *n*-hexane-isopropanol (9∶1, v/v) as the mobile phase. Fifteen racemic compounds were successfully separated on the chiral HPLC column, including seven racemic compounds for baseline separation, at a flow rate of 0.1 mL/min using 254 nm as the detection wavelength at 25 ℃. The separation of 10 benzene position isomers was successfully achieved, and eight of the benzene position isomers reached baseline separation. Experimental results showed that the chiral silica gel spheres possess sufficient mechanical strength and thermal stability, along with good chiral recognition ability and the ability to separate positional isomers. Compared with ordinary silica gel, these chiral silica gel spheres afforded better chiral separation and better separation of benzene position isomers, without further modification. The preparation of this chiral stationary phase also has the advantages of being cheap, convenient, and feasible.

手性是一种在生命系统中广泛存在的特征,是自然界的基本属性。在各个领域有着诸多应用。例如在药物方面,用于临床的1700~2000种合成药中,约有40%为外消旋体^[1]^。手性药物的左旋体和右旋体虽然具有相同的理化性质,但是它们的药理作用存在一定差异,甚至有相反的药理作用^[2]^。例如,苯并吗啡烷的两个对映体都有镇痛作用,但(+)-苯并吗啡烷服用后会成瘾,而(+)-苯并吗啡烷则不会。所以对手性化合物进行拆分是非常有必要的。

HPLC是目前使用范围广、拆分效率高的手性拆分方法之一^[3]^。HPLC在进行手性拆分的过程中起到核心作用的是色谱柱,其手性拆分能力取决于手性色谱填料,也称之为手性固定相(CSP)^[4]^。HPLC柱填料中主要的固定相材料是无机填料,主要包括硅胶、碳机制填料、氧化铝、氧化锆等^[5,6]^,其中硅胶占九成以上^[7]^。因此,研究手性硅胶就显得尤为重要。手性硅胶分为两大类,一类是无机手性硅胶^[8]^,另一类是在表面键合^[9]^或者涂覆^[10]^手性物质的硅胶,目前以后者占多数。但是这类固定相不耐高温,色谱柱寿命较短,受流动相种类限制大。无机手性硅胶在这些方面的限制小,因此无机手性介孔硅成为研究的前沿。经过长期的理论和实践总结,Unger等^[11]^给出了HPLC理想固定相的评价标准:无定型微粒填料不利于传质,会使操作压力增大,所以色谱柱填料最好为球形且粒径分布均匀的微粒。尤其是直径为2~5 μm球形微粒的优势更加明显,会比无定型微粒色谱柱的柱效更高。

各研究人员^[12,13,14]^分别合成了左手螺旋结构的手性向列相液晶薄膜、螺旋形貌和螺旋孔道介孔硅,以及用十二烷基硫酸钠为结构导向的螺旋介孔材料。袁黎明课题组^[15,16,17]^以这些为基础合成了片状手性向列相介孔硅,六方棱柱螺旋手性介孔硅以及短棒状手性无机介孔硅,但是这些报道全是无定型材料,并未见球形的全无机手性硅胶用作液相色谱手性固定相的研究中。

本文以无机球形介孔硅胶作为研究对象,将其用作高效液相色谱固定相,目的是制成一类制作简单便捷、可耐高温、可适应多种流动相并且有良好手性分离性能的新型手性固定相。主要研究工作是采用堆砌硅珠法^[18,19,20,21]^,用L-谷氨酸(L-Glu)为手性源,以硅溶胶为硅源合成脲醛树脂和二氧化硅混合小球,经高温煅烧除去有机部分获得无机手性硅胶球,手性来源于硅胶球自身的孔结构和骨架。然后将手性硅胶球填充到HPLC柱中,并在一定条件下,让L-Glu手性硅胶球固定相对手性外消旋异构体和常见的苯系位置异构体进行拆分,最终对15对外消旋体和10种苯系位置异构体有不同的拆分效果。

## 1 实验部分

### 1.1 仪器及材料

Elite P230Ⅱ高效液相色谱仪配有AT-330柱温箱(大连依利特公司); S-3000N扫描电子显微镜(日本Hitachi公司); JEM-2100透射电子显微镜(日本JEOL公司); Chirascan圆二色谱仪(英国Applied Photophysics公司); SX2-4-10马弗炉(上海意丰电炉有限公司); DJ-1大功率磁力搅拌仪(常州申光仪器有限公司); TDZ5-WS台式低速离心机(湖南湘仪实验室仪器开发有限公司);不锈钢液相色谱空柱(250 mm×2.0 mm)和1666型液相色谱装柱机(美国Alltech有限公司); DHG-9035A鼓风电热干燥箱(上海恒以科学仪器有限公司); ASAP2020 M+C氮气吸附仪(美国Micromeritics公司)。

L-谷氨酸(纯度>99%)、尿素(纯度≥99%)购于大连美伦生物公司;甲醛水溶液(纯度37%~40%)购于上海Adamas试剂公司;硝酸(纯度65%~68%)购于重庆川东化工有限公司;粗制硅胶购于中国青岛美高化工有限公司;硅溶胶纯度为30%,以及手性化合物:特罗格尔碱、吡喹酮、氨氯地平、3-苄氧基-1,2-丙二醇、苄氟噻嗪、1-苯丙醇、4-甲基-2-戊醇、2-氯丙酸、1,2-环氧己烷、氧化苯乙烯、3-羟基-2-丁酮、2-甲基环己酮、2-氯环己酮、2-甲基四氢呋喃-3-酮、异丙基缩水甘油醚纯度均>98%,均购于美国Sigma-Aldrich公司;位置异构体:*o*,*m*,*p*-氨基苯酚、*o*,*p*-氯苯酚、*o*,*m*,*p*-碘苯胺、*o*,*m*,*p*-甲苯胺、*o*,*m*,*p*-二硝基苯、*o*,*m*,*p*-氯苯胺、*o*,*m*,*p*-硝基苯胺、*o*,*m*,*p*-硝基苯酚、*o*,*m*,*p*-溴苯胺、*o*,*p*-硝基溴苯、*o*,*m*,*p*-苯二胺纯度均>97%,购于上海阿拉丁化学试剂公司。

### 1.2 以L-Glu为手性源的手性硅胶球材料的制备

取1 mmol L-Glu溶于20 mL硅溶胶中,加入1.0 g尿素,搅拌20 min。用1 mol/L HNO_3_将溶液pH值调至1.5,搅拌加入1.5 mL 37%甲醛水溶液,反应15 min,然后加入200 mL去离子水使反应停止,分别用水、甲醇、丙酮洗涤3次,于60 ℃干燥12 h。最后将其置于马弗炉,在550 ℃下高温煅烧6 h后降到室温,得到L-Glu手性硅胶球,制备过程见图1。

**图 1:**
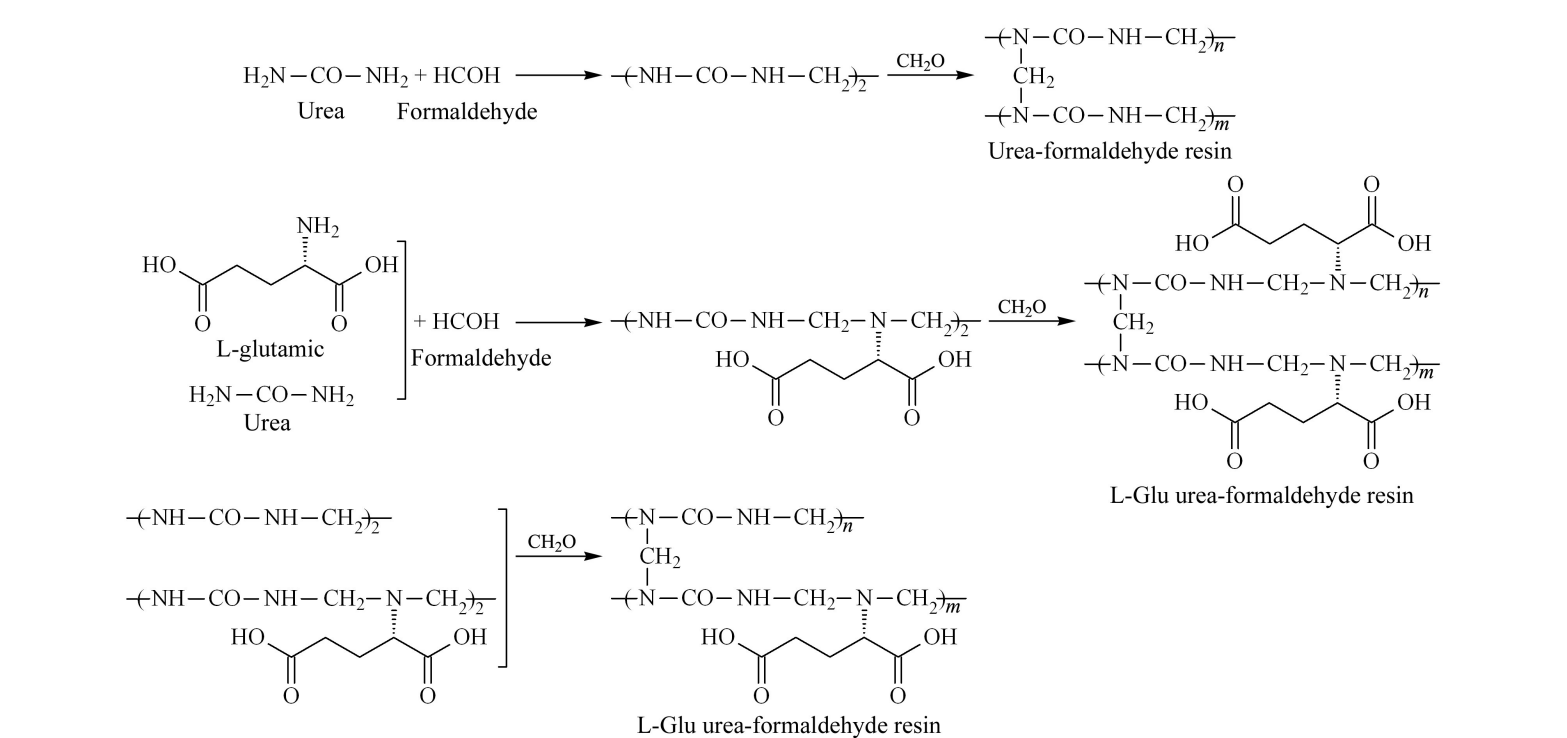
L-Glu、尿素和甲醛的缩聚反应

### 1.3 L-Glu手性硅胶球液相色谱柱的填充

取1.2 g干燥后的L-Glu手性硅胶球分散到23 mL正己烷-异丙醇(9∶1, v/v)溶液中。采用高压匀浆法装柱,制备色谱柱(250 mm×2.0 mm)。

### 1.4 色谱条件

流动相为正己烷-异丙醇(9∶1, v/v),流速为0.1 mL/min,紫外检测波长为254 nm,柱温为25 ℃。

## 2 结果与讨论

### 2.1 L-Glu手性硅胶球的形成原理

采用堆砌硅珠法,得到含有大量L-Glu的二氧化硅脲醛树脂球,经过洗涤、煅烧、浮选后得到L-Glu手性硅胶球。具体反应如下:

该反应由于L-Glu的加入,L-Glu中的-NH_2_基团会部分取代尿素中的-NH_2_基团参加反应,使缩聚产生的多聚体带有含手性碳原子的L-Glu。在多聚体相对分子质量不断增长的过程中,其会使硅溶胶中的二氧化硅纳米粒子逐渐团聚而最终沉降。而L-Glu具有一定的印迹以及诱导作用,在二氧化硅纳米粒子团聚时,所生成的产物具有一定的手性。

### 2.2 L-Glu手性硅胶球的表征

2.2.1 L-Glu手性硅胶球的元素分析

对L-Glu手性硅胶球进行元素分析,结果见表1。结果显示,L-Glu手性硅胶球中几乎不含C元素和N元素,和商业用粗制硅胶对比发现,N元素含量一致,L-Glu的H元素和C元素含量略多,但总体水平一致,说明L-Glu手性硅胶球被充分煅烧,合成中使用的氨基酸和生成的脲醛聚合物已经被灼烧完全,几乎不含有机部分,表明L-Glu手性硅胶球是由无机元素构成的材料。

**表 1:** L-Glu手性硅胶球的元素分析

Sample	N/%	C/%	H/%
L-Glu chiral silica sphere	<0.1	0.2-0.3	0.3
Crude silica gel	<0.1	<0.1	0.2

2.2.2 L-Glu手性硅胶球的红外光谱分析

分别对甲醛和尿素反应生成的脲醛树脂球、在硅溶胶中甲醛和尿素生成的脲醛树脂球、加入手性源L-Glu在硅溶胶中甲醛和尿素生成的L-Glu脲醛树脂硅胶球,以及最终经过烧制的L-Glu手性硅胶球,4种材料进行红外光谱分析(见图2)。

**图 2:**
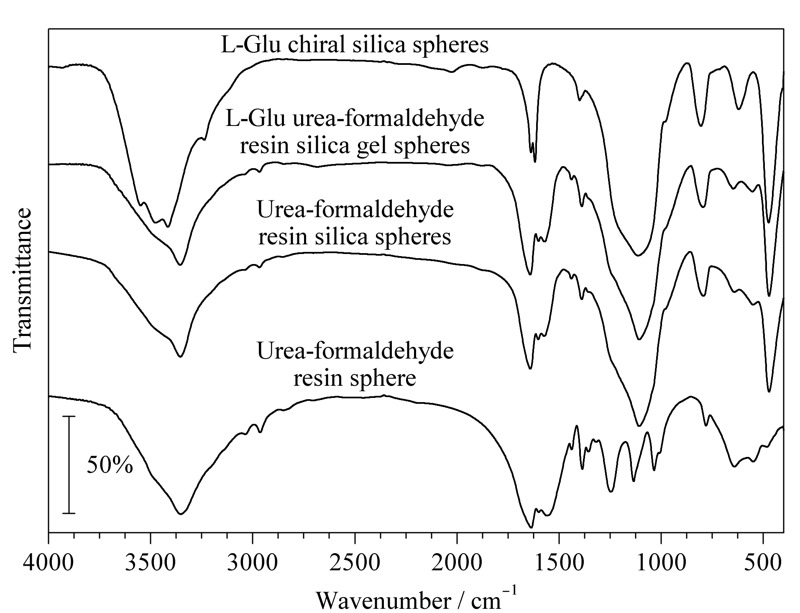
4种材料的红外光谱图

甲醛和氨基在水溶液中反应生成脲醛树脂球,可以看出在2900、1637和1246 cm^-1^处分别为C-H、C=O、C-N的特征吸收峰。反应环境换成硅溶胶后,出现明显的非晶体的硅吸收峰,包括471 cm^-1^处的Si-O-Si弯曲伸缩振动吸收峰,793 cm^-1^处的对称伸缩振动吸收峰和1107 cm^-1^处的反对称伸缩振动吸收峰。加了手性源的L-Glu脲醛树脂硅胶球因为红外显色基团,与脲醛树脂硅胶球没有明显的不同。经过烧灼的最终产物L-Glu手性硅胶球的红外光谱,保留了硅的特征峰,并且2900 cm^-1^左右峰消失,3500 cm^-1^左右的OH吸收峰凸显。

2.2.3 L-Glu手性硅胶球的扫描电镜分析

为进一步了解L-Glu手性硅胶球的形貌特征,对L-Glu手性硅胶球进行扫描电镜(SEM)和透射电镜(TEM)分析。从图3中可以看出,L-Glu手性硅胶球的SEM图呈现出较为规整无团簇的球形外观特征,粒径为3.0~4.5 μm,粒径分布较为均匀。硅胶球表面呈现出不光滑的小颗粒状,符合堆砌硅胶球法合成的硅胶球形貌特征。从TEM图可以看出,L-Glu手性硅胶球是由一颗一颗纳米硅胶球堆积而成,其孔径较为均一,由于是堆积孔,孔与孔之间的排列有交叉现象。

**图 3:**
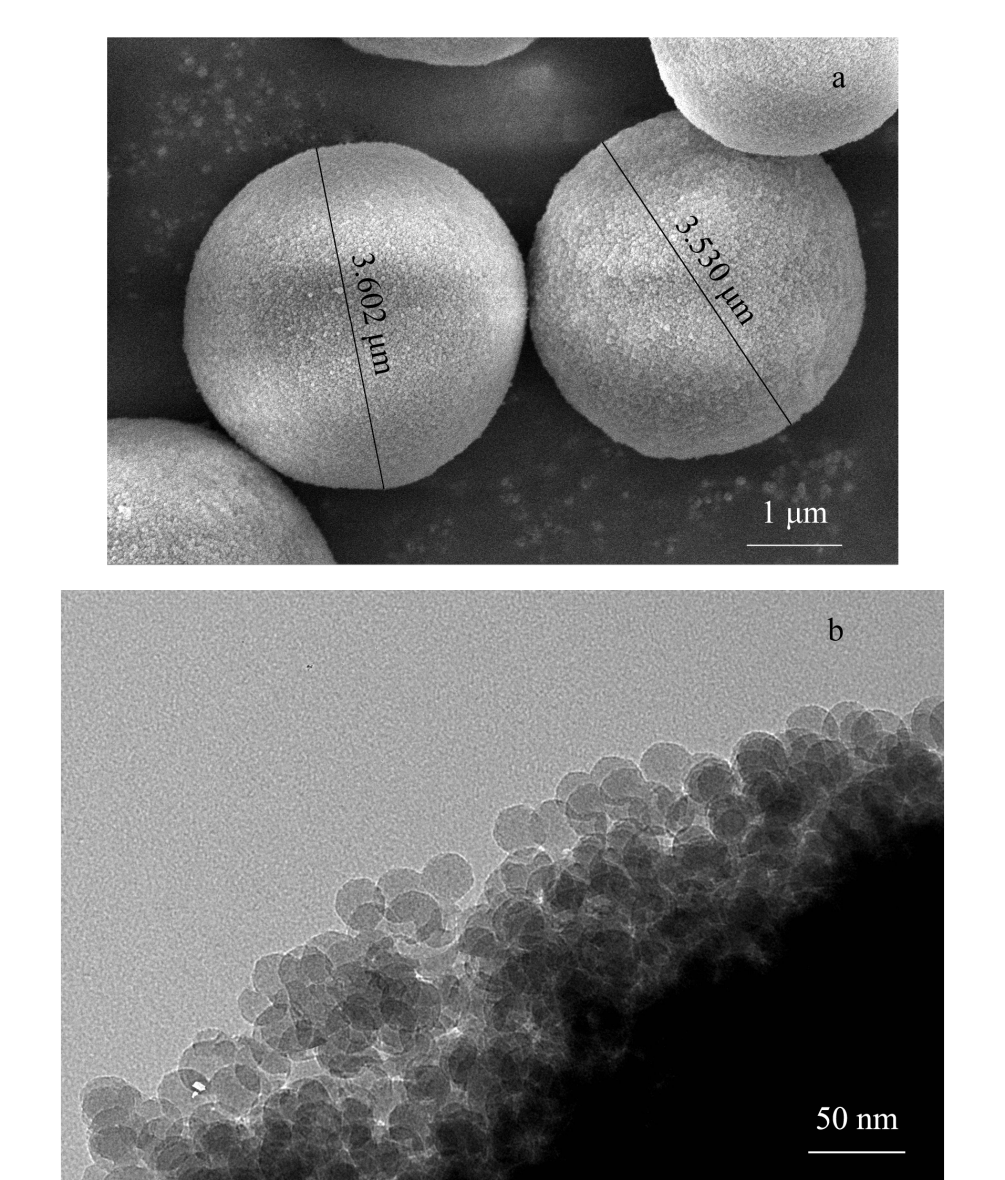
L-Glu手性硅胶球的扫描电镜和透射电镜图

2.2.4 L-Glu手性硅胶球的氮气吸附测试

对L-Glu手性硅胶球进行氮气吸附测试,使用标准的BET方程对吸附数据进行分析,氮气吸附脱附等温线和L-Glu手性硅胶球孔径分布曲线见图4。结果表明,L-Glu手性硅胶球的比表面积为117.844 m^2^/g,孔体积为0.411 cm^3^/g;通过等温线的BHJ分析,其平均孔径为12.312 nm。表明L-Glu手性硅胶球是一种中孔介孔硅胶,适合于一般有机化合物的分离。

**图 4:**
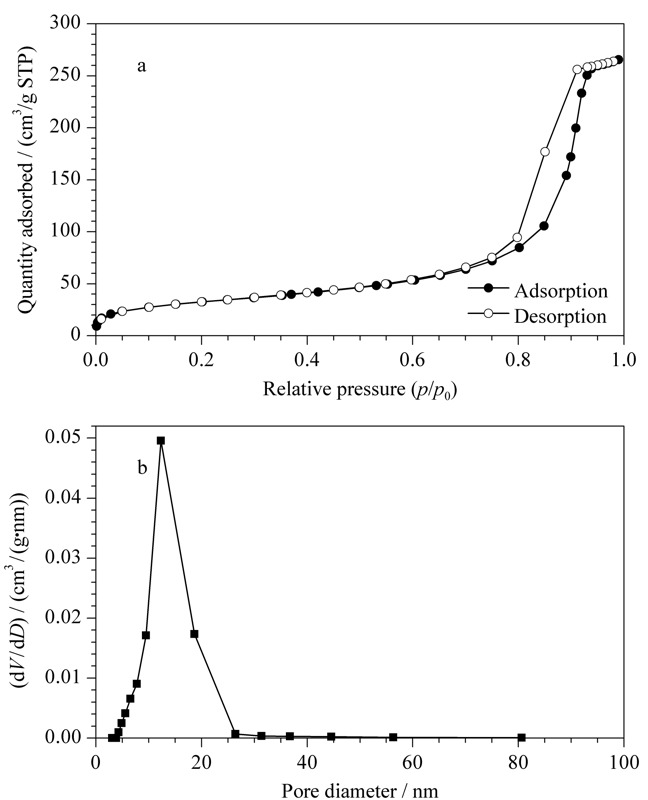
(a)氮气吸附脱附等温线和(b)L-Glu手性硅胶球孔径分布曲线

### 2.3 进样量对L-Glu手性硅胶球色谱柱分离性能的影响

为了研究进样量对L-Glu手性硅胶球固定相色谱柱分离性能的影响,在相同的条件下,取5、10、15、20 μL的2-甲基四氢呋喃-3-酮进行分析,以此研究进样量对对映体分离的影响(见图5)。

**图 5:**
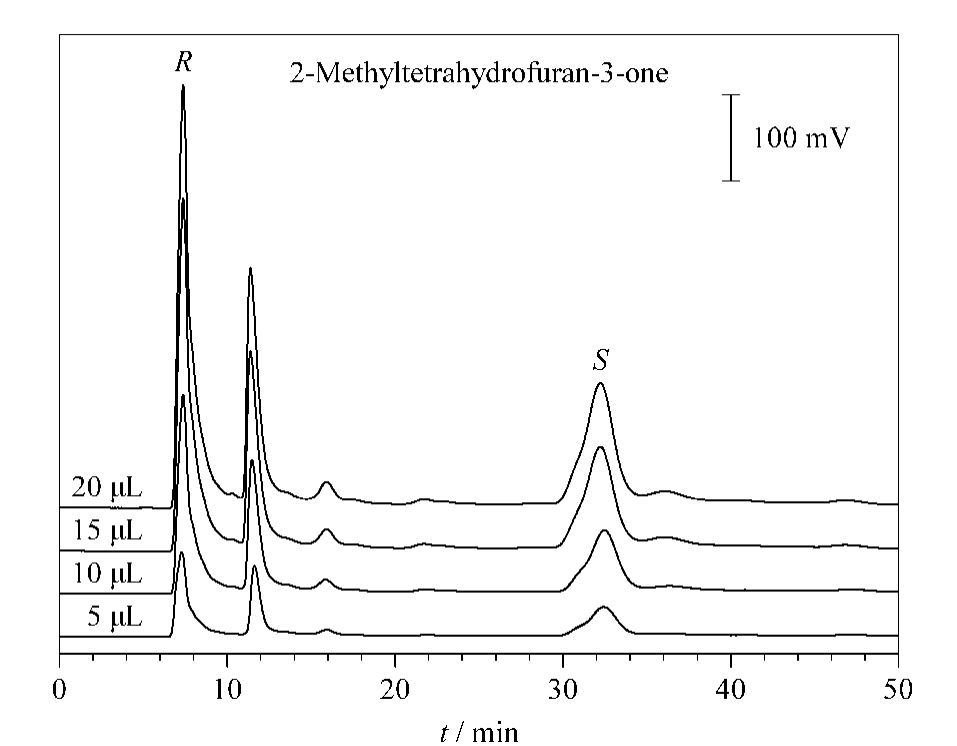
不同进样量下2-甲基四氢呋喃-3-酮的色谱图

随着进样量增加,2-甲基四氢呋喃-3-酮的峰面积也在增加,但是保留时间没有随着进样量不同而改变,选择性也保持稳定。因此在不同的进样量下,L-Glu手性硅胶球固定相色谱柱能保持稳定。

### 2.4 L-Glu手性硅胶球固定相色谱柱的重复性

为了研究L-Glu手性硅胶球固定相色谱柱的重复性,在相同的条件下,对*o*,*m*,*p*-甲苯胺进行5次进样,色谱图见图6。*o*,*m*,*p*-甲苯胺保留时间和峰面积的相对标准偏差(RSD)分别为0.89%和3.67%。该色谱柱重复性较好。

**图 6:**
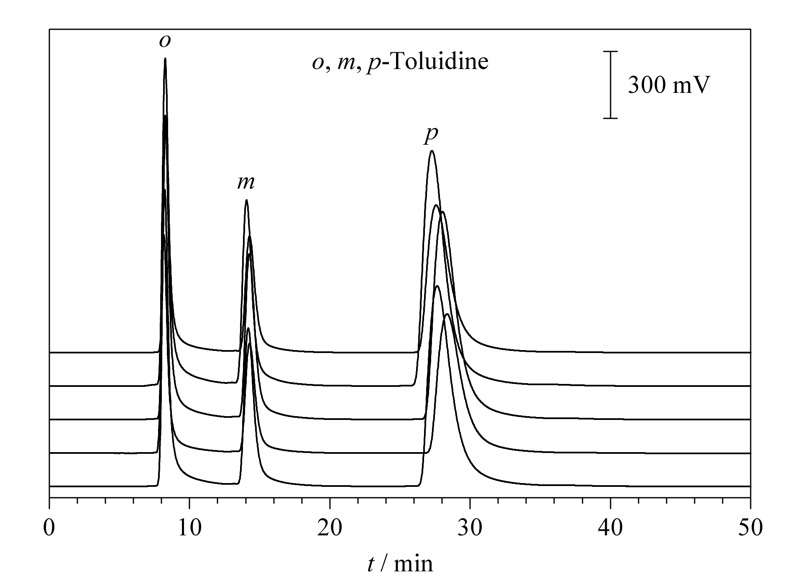
L-Glu手性硅胶球固定相的重复性

### 2.5 L-Glu手性硅胶球固定相对于手性外消旋体的拆分

为了检测L-Glu手性硅胶球固定相对于外消旋化合物的手性拆分能力,在一定条件下,用以L-Glu手性硅胶球为固定相的高效色谱柱对15种手性外消旋异构体进行拆分。

L-Glu手性硅胶球固定相分离效能采用保留因子(*k*)、分离因子(*α*)和分离度(*R*_s_)来评价,色谱柱死时间用三叔丁基苯测量。分离结果显示,L-Gul手性硅胶球固定相对15种外消旋体均有分离效果,其中特罗格尔碱、吡喹酮、3-苄氧基-1,2-丙二醇、1,2-环氧己烷、3-羟基-2-丁酮、2-甲基四氢呋喃-3-酮、异丙基缩水甘油醚7种外消旋手性异构体能够达到基线分离。表2是L-Glu手性硅胶球固定相对15种外消旋物质的拆分数据。

**表 2:** L-Glu手性硅胶球色谱柱对15种外消旋体的拆分结果

Racemate	k_1_	k_2_	α	R_s_
Troger’s base	0.21	1.42	6.45	4.70
Praziquantel	0.24	3.58	14.93	8.70
Amlodipine	10.02	11.46	1.14	0.74
3-Benzyloxy-1,2-propanediol	0.27	3.94	14.47	7.44
Bendroflumethiazide	1.98	4.37	2.21	0.03
1-Phenyl-1-propanol	0.26	0.50	1.92	0.84
4-Methyl-2-pentanol	0.22	0.49	2.22	1.35
2-Chloropropionic acid	0.24	0.40	1.68	0.59
1,2-Epoxyhexane	0.20	0.47	2.32	1.56
Styrene oxide	0.22	0.49	2.20	0.86
3-Hydroxy-2-butanone	0.20	1.40	9.16	7.11
2-Methylcyclohexanone	0.21	0.44	2.12	1.14
2-Chlorocyclohexanone	0.15	0.50	3.23	1.40
2-Methyltetrahydrofuran-3-one	0.20	0.92	4.53	3.90
Glycidyl isopropyl ether	0.20	1.64	8.07	7.90

*k*: retention factor; *α*: separation factor; *R*_s_: resolution.

L-Glu手性硅胶球是一种手性介孔硅材料。L-Glu为手性硅胶提供手性环境,使其具有手性孔道和骨架,让原本没有分离性质差异的对映体产生分离差异,从而达到分离效果;多孔结构也让外消旋体与其内壁上的作用位点的接触面增大,使外消旋体分配系数增大,提高了分离效率。手性固定相和对映体间的氢键作用对对映体也起到了拆分作用,如上述外消旋体中氨氯地平有H-N键、H-O键,3-苄氧基-1,2-丙二醇有H-O键等,除此之外偶极相互作用力以及范德华力也对对映体的拆分起到了作用。

### 2.6 L-Glu手性硅胶球固定相对于苯系位置异构体的拆分

为了检测L-Glu手性硅胶球固定相对于苯系位置异构体的拆分能力,在一定的条件下,用L-Glu手性硅胶球固定相色谱柱对11种常见的苯系位置异构体进行分离。分离结果表明,除*o*,*m*,*p*-苯二胺外,L-Glu手性硅胶球固定相对其余10种苯系位置异构体均有分离效果,对于*o*,*m*,*p*-氨基苯酚、*o*,*p*-氯苯酚、*o*,*m*,*p*-碘苯胺、*o*,*m*,*p*-甲苯胺、*o*,*m*,*p*-二硝基苯、*o*,*m*,*p*-氯苯胺、*o*,*m*,*p*-硝基苯酚、*o*,*m*,*p*-溴苯胺达到基线分离。表3是10种苯系位置异构体的分离数据。

**表 3:** L-Glu手性硅胶球固定相对于苯系位置异构体的拆分结果

Sample	α_1,2_	α_2,3_	R_s1,2_	R_s2,3_
o,m,p-Aminophenol	2.21	2.09	5.28	6.03
o,p-Chlorophenol	1.82	/	2.65	/
o,m,p-Iodoaniline	3.51	1.49	3.73	1.65
o,m,p-Toluidines	4.59	2.81	5.08	6.67
o,m,p-Dinitrobenzene	2.98	3.19	1.65	5.10
o,m,p-Chloroaniline	2.89	1.55	2.73	1.59
o,m,p-Nitroaniline	2.41	1.73	3.54	4.23
o,m,p-Nitrophenol	1.71	1.32	0.66	0.55
o,m,p-Bromoanilines	2.98	1.46	3.80	1.59
o,p-Nitrobromobenzene	1.35	/	0.34	/

*α*_1,2_: the separation factor of the first and second peaks; *α*_2,3_: the separation factor of the second and third peaks; *R*_s1,2_: resolution of the first and second peaks; *R*_s2,3_: resolution of the second and third peaks; /: no corresponding drug.

苯系位置异构体邻位、对位和间位分子的长宽比和体积大小不一样,所以在固定相上的受力也不一样,导致其的保留时间不同,从而使位置异构体能够分离。L-Glu手性硅胶球的多孔结构让苯系位置异构体与其内壁上的作用位点的接触面增大,使苯系位置异构体分配系数增大,提高了分离效率。除此之外,某些异构体如氨基苯酚、硝基苯酚等的邻位异构体形成了分子内氢键也对异构体的拆分起到了作用。L-Glu手性硅胶球固定相表现出了在分离苯系位置异构体上的可能性。

## 3 结论

通过堆砌硅珠法,以L-Glu为手性源,制备了L-Glu手性硅胶球。这种手性硅胶球是一种无机手性硅胶,它弥补了部分其他手性硅胶不耐腐蚀、柱子寿命较短、受流动相种类限制大等缺陷。将L-Glu手性硅胶球用作手性固定相,成功拆分了15种外消旋化合物和10种苯系位置异构体,且对部分外消旋化合物和苯系位置异构体较好的拆分效果。实验表明,L-Glu手性硅胶球在手性分离方面具有良好的可行性,或是一种低成本、制备便捷的手性无机硅胶固定相。
